# Socioeconomic inequalities and diabetes: A systematic review from Iran

**DOI:** 10.1186/s40200-015-0135-4

**Published:** 2015-02-25

**Authors:** Niloofar Peykari, Shirin Djalalinia, Mostafa Qorbani, Sahar Sobhani, Farshad Farzadfar, Bagher Larijani

**Affiliations:** Non-communicable Diseases Research Center, Endocrinology and Metabolism Research Institute, Tehran University of Medical Sciences, 4th floor, No 4, Ostad Nejatollahi St, Enqelab Ave, Tehran, Iran; Endocrinology and Metabolism Research Center, Endocrinology and Metabolism Research Institute, Tehran University of Medical Sciences, 5th floor, Shariati Hospital, Kargar St, Tehran, Iran; Development of Research & Technology Center, Deputy of Research and Technology, Ministry of Health and Medical Education, Tehran, Iran; School of Medicine, Community Medicine Department, Alborz University of Medical Sciences, Karaj, Iran

**Keywords:** Diabetes, Socioeconomic factors, Iran

## Abstract

Socioeconomic factor is a determinant of health may contribute to diabetes. We conducted a systematic review to summarizing evidences on associations between socioeconomic factors and diabetes in Iranian population. We systematically searched international databeses; ISI, PubMed/Medline, Scopus, and national databases Iranmedex, Irandoc, and Scientific Information Database (SID) to retrieve relevant articles to socioeconomic factors and diabetes without limitation on time. All identified articles were screened, quality assessed and data extracted by two authors independently.

From 74 retrieved articles, 15 cases were relevant. We found increased diabetes prevalence among female sex, over 50 years’ old age, illiterate population, retired status, unemployed, urban residents, and low economic status. There was a negative association between social capital and diabetes control. Diabetes complications were more frequent in upper age group, higher education levels and low income populations.

Socioeconomic factors were associated with diabetes that leads to inequality. Improving modifiable factors through priority based interventions helps to diabetes prevention and control.

## Introduction

Diabetes is responsible to 1,281,340 death in 2010 across the world, and its’ attributed mortality has doubled compared to 1990 [1,2]. Surprisingly, about one million death due to diabetes occurred in developing countries [1]. Dietary risk is the leading risk factor in this area and it’s not worthy that, dietary, behavioral and metabolic risk factors are the main risk factors of diabetes [1,3].

In Iran, during the same time period, this considerable problem had remarkable increase and has become the leading cause of death (14.8 per 100,000) in 2010 [1,4]. More than half of premature deaths are due to conditions that could be prevented or treated through effective policies and interventions [5-8].

Although the socioeconomic factors (SEFs) and health status have not straightforward associated, it is inevitable that, various social and economical factors have direct or indirect impact on the health status [9]. Diabetes prevalence is affected by socioeconomic factors [2,10-12]. Moreover, access to health care, treatment choices, and control recommendations are affected by SEFs [13,14]. Thus the Health inequality is important challenge that should be considered in lower socioeconomic groups [15,16]. This requires an active engagement of health providers and policy makers and researchers should be shift from descriptive studies to interventional studies [17].

In particular, diabetes was the subject of the 25 × 25 non-communicable disease mortality reduction target [18,19]. The commitment to this target require consideration various aspect of problem. Several studies showed association between some SEFs and diabetes incidence and revealed that low socioeconomic status is a barrier in access to diabetes care in developing countries [13,20-22].

Within countries, inequality assessment and estimate the effect of socioeconomic factors on diabetes could provide information to priority setting and planning for effective interventions to inequity reduction and socioeconomic modification to decrease the diabetes burden and achievement the 25 × 25 targets [23].

The scarcity of related studies in Iran, motivate us to conduct a systematic review. The aim of this systematic review is to describe the cross-sectional association between socioeconomic factors and diabetes in Iranian population.

## Methods

### Terms’ Definition

Diabetes defined as “A heterogeneous group of disorders characterized by hyperglycemia and glucose intolerance”[24] and SEFs differentiate the individual or group within the social structure [25]. For each of SEFs, classic definition presented in Table 1 [25].

**Table 1:** **Socioeconomic factors’ definitions**

**Socioeconomic factors**	**Classic definition**
**Age group**	Persons classified by age from birth (INFANT, NEWBORN) to octogenarians and older (AGED, 80 AND OVER).
**Sex**	The totality of characteristics of reproductive structure, functions, PHENOTYPE, and GENOTYPE, differentiating the MALE from the FEMALE organism.
**Educational level**	Educational attainment or level of education of individuals.
**Marital status**	A demographic parameter indicating a person’s status with respect to marriage, divorce, widowhood, singleness, etc.
**Occupation**	Crafts, trades, professions, or other means of earning a living.
**Income**	Revenues or receipts accruing from business enterprise, labor, or invested capital.
**Residence characteristics**	Elements of residence that characterize a population. They are applicable in determining need for and utilization of health services.
**Urbanization**	The process whereby a society changes from a rural to an urban way of life. It refers also to the gradual increase in the proportion of people living in urban areas.

### Data sources and search strategy

We carried out a systematic search among three international databases; PubMed/Medline, Institute of Scientific Information (ISI), Scopus and three national databases; IranMedex, Scientific Information Database (SID), and Irandoc. To obtain the most comprehensive and efficient results, we searched these data sources using Medical Subject Headings (MeSH) terms, Emtree, and related key words. Moreover, in national databases, we considered related Persian key words in addition to English search terms. To achievement additional studies, we reviewed manually the references and citations of relevant articles. Our search strategy present in Table 2. All kinds of studies which performed in *Iran* related to diabetes and socioeconomic inequalities were included. There was no limitation on age, time and language.

**Table 2:** **The Search strategy**

**Domain**	**Search strategy**
Diabetes	(“diabetes mellitus”[MeSH Terms] OR (“diabetes”[All Fields] AND “mellitus”[All Fields]) OR “diabetes mellitus”[All Fields]) AND “[Mesh] OR (”[All Fields] AND (“diabetes mellitus”[MeSH Terms] OR (“diabetes”[All Fields] AND “mellitus”[All Fields]) OR “diabetes mellitus”[All Fields]) AND ((“medical subject headings”[MeSH Terms] OR (“medical”[All Fields] AND “subject”[All Fields] AND “headings”[All Fields]) OR “medical subject headings”[All Fields] OR “mesh”[All Fields]) AND Terms[All Fields]) AND “diabetes mellitus”[MeSH Terms] OR (“diabetes”[All Fields] AND “mellitus”[All Fields]) OR “diabetes mellitus”[All Fields] OR “diabetes”[All Fields]
Socioeconomic factors	(((((((“Socioeconomic Factors”[Mesh] OR “Poverty”[Mesh]) OR “Social Class”[Mesh]) OR “Educational Status”[Mesh]) OR “Employment”[Mesh]) OR “Family Characteristics”[Mesh]) OR “Income”[Mesh]) OR “Occupations”[Mesh]) OR “Social Conditions”[Mesh] OR “Standard of Living”[All Fields] OR “living standard”[All Fields] OR “land tenure”[All Fields] OR “High-Income Population”[All Fields] OR “High Income Population”[All Fields] OR (“socioeconomic factors”[MeSH Terms] OR (“socioeconomic”[All Fields] AND “factors”[All Fields]) OR “socioeconomic factors”[All Fields] OR “inequality”[All Fields]) OR (“socioeconomic factors”[MeSH Terms] OR (“socioeconomic”[All Fields] AND “factors”[All Fields]) OR “socioeconomic factors”[All Fields] OR”inequalities“[All Fields])
Geographic area	(((“iran”[MeSH Terms] OR “iran”[All Fields]) OR iranian[All Fields] OR I.R.Iran[All Fields] OR “persia”[MeSH Terms]) OR ((“iran”[MeSH Terms] OR “iran”[All Fields]) OR iranian[All Fields] OR I.R. Iran[All Fields] OR persia[Title/Abstract])) OR ((“iran”[MeSH Terms] OR “iran”[All Fields]) OR iranian[All Fields] OR I.R. Iran[All Fields] OR persia[Text Word])

### Study selection

At the first stage of study selection process, the reviewers read the titles and abstracts. If they didn’t related to our search objectives, these articles excluded. Studies in non-Iranian population and interventional studies were excluded. If some studies focus on low socioeconomic groups such as slums or considered only high socioeconomic groups such as special high income business, they were excluded from our systematic review because of bias control and intention to normal population.

We included original articles. To achieve comprehensive results, review articles considered for backward and forward assessment of their references and citations. Qualitative studies, letters, editorial and all of other article types were excluded.

In second stage, for all of included articles, full texts reviewed by two independent reviewers for quality assessment and data extraction. In cases of difference between reviewers, the third reviewer resolved discrepancy.

### Quality assessment and data extraction

For quality assessment of included articles, we used the critical appraisal skills program (CASP) checklists [26]. The assessment conducted by two independent reviewers. Discrepancies have resolved by a third reviewer.

Data extraction sheet was designed including two main parts of; study characteristics’, and extracted data. The study characteristics sheet contained; article’s specifications, corresponding author’s characteristics, study’s method, and study’s quality scale. The data extraction sheet also, contains detailed information on diabetes prevalence in various SEF status, Odds ratio (OR), main outcome and suggestions.

### Data synthesis

We systematically categorize results according to various aspects of outcome. So in this review, each aspect of results summarized and presented in different tables.

### Ethical consideration

As this study is systematic review, it didn’t need to ethical approval. Regarding ethical consideration in this study, we cited all scientific documents.

## Results

**Figure 1:**
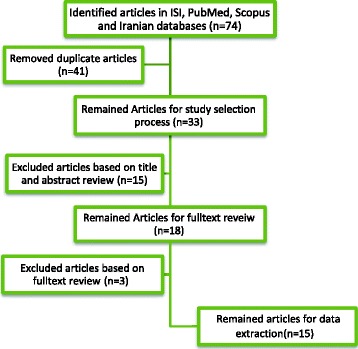
**Study selection process.**

Considering inclusion and exclusion criteria, 15 articles that met eligible criteria remained for data extraction (Figure 1).

### Descriptive findings

All of searched articles were in English or Persian language. Although, we haven’t limit the search strategy on certain time; retrieving articles were between 1998 and 2014. Included articles were published in 2008-2014 time period and their study year was between 2001 and 2012.

All included studies were Cross sectional. Eight articles were population based study and the others were clinical and hospital based. One of the included articles was at national level and the others were provisional. Forty percent of studies’ participants were from general population and the others were diabetic patients (except one study witch targeted overweight and obese persons). In general, these results are attributed to 13,711 diabetic patients from included studies. Excluding other than one study that focused on female sex, the remained studies covered both sex. According to content of papers, we present results in five domains; a) Inequality and diabetes, b) diabetes prevalence, c) diabetes control, d) diabetes complications, and e) remained information on other related subjects. The details of included studies presented in Table 3.

**Table 3:** **Socioeconomic factors and diabetes**

**a)Inequality index and diabetes**											
**No**	**Reference**	**Study design and Setting**	**Study year**	**Participants and their recruitment**	**Sex**	**Age (Year)**	**Inequality Assessment Method**	**Concentration Index measure(±SE) related to Diabetes**		**Main Conclusion**	**Suggestion**
**1**	**Emamian MH, et al. 2011[** 27 **]**	**Cross sectional study, Population based/** ***Shahroud***	**2005**	**General Population, Random sampling/ n=1000(5.3% diabetic patients)**	**Both/Female (50%)**	**15-64**	**Concentration Index**	**Both**	**0.044±0.072**	**Concentration curve difference from the line of equality for diabetes isn’t significant.**	**Especial attention to poverty alleviation in upper age groups according to the role of age and low economic status in NCDs' occurrence**
								**Female**	**0.074±0.09**	**Age, governmental employee, being unmarried, residence in rural area and low economic status are the most important factors which influence on NCDs' inequalities.**	
								**Male**	**0.001 ± 0.115**		
**b)Diabetes prevalence**											
**No**	**Reference**	**Study design and Setting**	**Study year**	**Participants and their recruitment**	**Sex**	**Age (Year)**	**Socioeconomic Factors**	**OR (95% CI)**		**Main Conclusion**	**Suggestion**
**2**	**Maddah, M. 2010[** 28 **]**	**Cross sectional study, Population based/** ***Gilan***	**2007**	**General Population, random sample/n=9046(10.8% diabetic patients)**	**Female**	**≥25 years**	**Age/Educational levels/living areas**	**Diabetes and SEF**		**Increasing age in women associated with diabetes and in women living in low income areas, diabetes is more prevalent. In addition, diabetes is more common in the lowest educational level.**	**Prevention of diabetes in Iranian women especially in low socioeconomic level**
								**Age**	**0.9 (0.8–0.9)**		
								**Educational levels (years)<5**	**1.36(0.51-3.65)**		
								**Living in low income area**	**1.43 (1.05-1.94)**		
**3**	**Golozar A. et al. 2011[** 29 **]**	**Cross sectional study, Population based/** ***Golestan***	**2007**	**Diabetic Patients/Systematic clustering/n=3453**	**Both/Female (68.08%)**	**30 -87**	**Gender**	**Diabetes and SEF**		**The diabetes prevalence increased 21% for every 10-year increase in age. In urban area, non-Turkmen ethnicity, low economic status and illiterate persons, diabetes is more prevalent. Socioeconomic status was inversely associated with diabetes prevalence.**	**Improving DM awareness, improving general living conditions, and early lifestyle modifications**
							**Educational level/**	**Female**	**1.62(1.5-1.74)**		
							**Economic status/**	**Illiterate**	**1.26(1.16-1.36)**		
							**Residence**	**Low economic status**	**1.52(1.41-1.64)**		
								**Urban**	**1.56 (1.45-1.69)**		
**4**	**Azimi-Nezhad, M.et al. 2008[** 31 **]**	**Cross sectional study, Populatio5n based/** ***Khorasan***	**2008**	**General Population, cluster-stratified sampling/n=3778 (5.5% diabetic patients)**	**Both/Female (50 %)**	**15-64**	**Gender/Age/Educational level/Occupation/Marital status/Residence**	**Diabetes and SEF**		**Diabetes is prevalent in urban areas, female persons, and retirees and unemployed. There was no association between education, marital status and diabetes.**	**Primary prevention by lifestyle interventions especially in urban area. The preventive strategies should be based on the affective factors**
								**Female**	**1.15(0.86-1.52)**		
								**Age,≥ 50**	**3.13(2.34-4.17)**		
								**Married**	**0.91(0.59-1.39)**		
								**Illiterate**	**1.19 (0.88-1.6)**		
								**Retired**	**2.41(1.52-3.82)**		
								**Unemployed**	**2.05(1.13-3.72)**		
								**Urban**	**2.73(1.89–3.92)**		
**5**	**Veghari, Gh. et al. 2010[** 30 **]**	**Cross sectional study, Population based/** ***Golestan***		**General Population, stratified sampling/n=1998(8.3 diabetic patients)**	**Both/Female (49.9%)**	**25- 65**	**Gender**	**Hyperglycemia and SEF**		**The diabetes is more prevalent in women than men. Age > 55years, illiteracy, and residence in urban area have OR>1 with Hyperglycemia.**	**Screening and education of DM patients.**
							**Age**	**Female**	**1.48(1.07-2.05)**		
							**Educational level/**	**Age ,≥ 55**	**3.31 (2.38-4.60)**		
							**Economic status/**	**Illiterate**	**1.37 (0.99-1.90)**		
							**Residence**	**Urban**	**1.52 (1.10-2.10)**		
								**Low and medium economic status**	**1.16 (0.46-2.91)**		
**6**	**Shahraki, M. et al. 2012[** 32 **]**	**Cross sectional study, Clinical Based/** ***Zahedan***	**2012**	**Overweight and obese women/Non random sampling n=811**	**Female**	**20–60**	**Age/Educational level**	**FBS levels and SEF**		**Age and education significantly associated with FBS levels.**	**Encourage to physical activity and healthy diet among women**
								**≤ Age50**	**3.8 (1.798.45)**		
								**Educational level≤12**	**1.9 (1.25 -3.15)**		
**c) Diabetes control**											
**NO**	**Reference**	**Study design and Setting**	**Study year**	**Participants and their recruitment**	**Sex**	**Age (Year)**	**Socioeconomic Factors**	**OR (95% CI)**		**Main Conclusion**	**Suggestion**
**7**	**Farajzadegan, Z. et al. 2013[** 33 **]**	**Cross sectional study, Population based/** ***Isfahan***	**2010**	**Diabetic patients, random sampling/n=120**	**Both/Female (81.6%)**	**≥30 years**	**Gender**	**Diabetes control and SEF**		**There was a significant negative correlation between total social capital score and diabetes control. Also empowerment and political action and trust and solidarity dimensions and the level of HbA1c have negative correlation.**	**The creation of social capital to improve diabetes control**
							**Occupation**	**Female**	**1.56(0.61-4.00)**		
								**Housewife**	**2.22(0.95-5.19)**		
								**Retired**	**3.22(0.62-16.65)**		
**8**	**Mirzazadeh, A. et al. 2009[** 36 **]**	**National Cross sectional study, population-based /** ***Iran***	**2005**	**General population, random sampling/n=89 (5.6% diabetic patients)**	**Both/Female**	**25–64**	**Age**	**Diabetes control and SEF**		**Inhabitants in rural areas controlled diabetes better than who lived in an urban area. Also, control of the fasting plasma glucose level was better in younger diabetic patients.**	**More attention to elderly diabetic patients(Particularly those in urban areas)**
							**Gender**	**Female**	**1.13(0.97-1.32)**		
							**Marital status**	**Age>55**	**5.29(3.42-8.18)**		
							**Educational level**	**Married**	**1**		
							**Residence**	**Single**	**0.94(0.59-1.54)**		
								**Illiterate**	**1**		
								**literate**	**1.11(0.93-1.32)**		
								**Urban**	**1.39(1.16-1.67)**		
**9**	**Esmaeil-Nasab, N. et al. 2010[** 35 **]**	**Cross sectional study, Clinical Based/Sanandaj**	**2008**	**Type 2 Diabetic patients/random sampling/n-411**	**Both/Female (74.5%)**	**>25**	**Gender**	**HgA1c<6 and SEF**		**There was significant correlation between HgA1c and sex, age, educational level and occupation. OR between age and HgA1c was 1.2.**	**----**
							**Educational level**	**Male**	**2.46(1.37-4.42)**		
							**Occupation**	**Illiterate**	**3.42(2.16-5.40)**		
								**Unemployed**	**2.59 (1.27-5.26)**		
**10**	**Jahanlou, A. S. et al. 200[** 37 **]**	**Cross sectional study, Clinical Based/** ***Bandarabas***	**2007**	**Diabetic patients/Non random sampling 4=140**	**Both/Female (67.5%)**	**27-72**	**Educational level**	**HbA1c level and Educational level**		**Illiteracy and HbA1c >7 have OR (1.24) but Literacy level does not have a role in glycemic control.**	**Promotion health literacy**
								**Illiterate**	**1.24 (0.72-2.14)**		
								**>7 years schooling**	**1.12(0.64-1.94)**		
**d) Diabetes complications**											
**NO**	**Reference**	**Study design and Setting**	**Study year**	**Participants and their recruitment**	**Sex**	**Age (Year)**	**Socioeconomic Factors**	**Association**		**Main Conclusion**	**Suggestion**
**11**	**Tol, A. et al. 2013[** 38 **]**	**Cross sectional study, Clinical Based/** ***Isfahan***	**2009**	**Diabetics Patients, Random sampling /n=384**	**Both/Female (47.9%)**	**25-99**	**Age/ Educational level/Incom**	**Relation between number of complications in diabetics patients and SEF**		**Three complications in the age group of 60 to 70 years old were more prevalent. Three complications in higher education levels were seen. The highest numbers of complications were among housewives and retired people. Most diabetic patients with complications were in the income group of less than 7200 $ per year.**	**Applying the supportive resources and strategies**
								**Age/**	**Sig (P<0.001)**		
								**Educational level/**	**Sig (P<0.001)**		
								**Income**	**Sig (P<0.001)**		
**12**	**Tol, A. et al. 2012[** 39 **]**	**Cross sectional study, Hospital Based/** ***Tehran***	**2010**	**Type 2 diabetic patients with complications/Non random sampling/n=450**	**Both / Female (46%)**	**≥25 years**	**Gender**	**Relation between number of complications in diabetics patients and SEF**		**Complications' frequency demonstrated significant relation with sex (female), age, educational level, type of occupation, and social class. The majority of patients (54.2%) belonged to low income group.**	**Empowering diabetic patients**
							**Age**	**Female**	**Sig (P<0.001)**		
							**Educational level/**	**Age**	**Sig (P<0.001)**		
							**Occupation/**	**Educational level**	**Sig (P<0.001)**		
							**Marital Status**				
							**Family Annual**	**Occupation**	**Sig (P<0.001)**		
							**Income**	**Marital status**	**Non Sig**		
								**Family Annual Income**	**Sig (P<0.001)**		
**13**	**Rahimian Boogar, I. et al. 2011[** 40 **]**	**Cross sectional study, Clinical Based/** ***Tehran***	**2010**	**Type 2 diabetic patients, convenience sampling/n=246**	**Both/Female (55.6%)**	**28-57**	**Gender**	**CVD Probability in diabetes patients and SEF (Odds ratio)**		**Sex and age of onset of diabetes are associated with cardiovascular complications among diabetic patients.**	**Planning preventive intervention for diabetes**
							**Age**	**Male**	**1.79(0.99-3.22)**		
							**Quality of life**	**Age of onset of diabetes(<45)**	**1.13(0.63-2.03)**		
							**Self management**				
**e) Other related subjects to diabetes**											
**No**	**Reference**	**Study design and Setting**	**Study year**	**Participants and their recruitment**	**Sex**	**Age (Year)**	**Socioeconomic Factors**	**OR (95% CI)**		**Main Conclusion**	**Suggestion**
**14**	**Shirani, S.et al. 2009[** 41 **]**	**Cross sectional study, Population based/** ***Isfahan, Najafabad, Arak***	**2001**	**General Population, Random sampling/ n=12514 (5.6% diabetic patients)**	**Both/Female (51%)**	**≥19 years**	**Gender**	**Awareness of Diabetes AND SEF**		**Female sex, age > 30 years, educational levels under diploma, retired situation, and married status have OR>1 with awareness of diabetes.**	**Community-based intervention programme/Public health measuring**
							**Age**	**Female**	**2.15 (0.53-7.74)**		**Public health measuring**
							**Educational level/**	**Age, ≥ 60**	**6.23 (2.14–18.11)**		
							**Occupation/**	**Illiterate**	**1.4 (0.56–3.5)**		
							**Marital status/**	**Unemployed**	**0.92 (0.37-2. 30)**		
							**Residence**	**Retired**	**1.06(0.46–2.44)**		
								**Married**	**1.26 (0.77–2.06)**		
								**Urban**	**0.96 (0.66–1.40)**		
**15**	**Najibi N, et al. 2013[** 42 **]**	**Cross sectional study, Clinical Based/** ***Fars***	**2011**	**Type 2 Diabetic patients/Random sampling/n=135**	**Both/Female (73.3%)**	**30-55**	**Economic status/Income/Family size/Number of childres**	**Food insecurity and SEF**		**Food insecurity was significantly associated with economic status, education level, income, having child under 18 years of age, family size, and number of children ,but there was not a significant relationship between food Insecurity and occupation, marital status.**	**Economic status promotion**
								**Economic**	**0.22(−0/57-0/08) status**		
								**Income**	**0.19(0/07-0/54)**		
								**Family size**	**3.9(1/53-9/94)**		
								**Number of children**	**3.5(1.23-9.97)**		

### Inequality and diabetes

Our systematic review revealed that, in Iran, only one study considered inequality assessment index about diabetes. Based on first run of non-communicable disease surveillance study’ data (STEPs study, 2005) in Shahroud, concentration index for diabetes was (–0.044) for both sex [27]. It showed that concentration index for female sex was negative and it was positive in male sex [27].

#### Diabetes prevalence

Five papers have assessed the association of SEFs and diabetes prevalence [28-32]. Female sex associated with diabetes and related odds ratio (OR) has reported 1.15, 1.48 and 1.62 among three studies [29-31]. They also reported the positive association between age and diabetes. Fasting blood sugar (FBS) levels and age (more than 50 years old) have odds ratio more than three [30-32]. These studies concluded increasing age especially in women associated with diabetes.

In addition, educational level significantly associated with Fasting Blood Sugar (FBS) levels [32]. According to these results, diabetes is more prevalent in illiterate persons (OR > 1) [29-31]. Also, two studies in Gilan and Zahedan demonstrated educational level less than five year and under diploma has respectively; OR: 1.36 and 1.9 comparison with upper levels of education [28,32].

Among different occupational status, retired status and unemployment had significant association with diabetes (respectively; OR: 2.41 and OR: 2.05) [31]. All ORs were adjusted for age and gender. Considering the residence place, living in urban area is associated with diabetes prevalence (OR: 1.52, 1.56, and 2.73). Diabetes is more prevalent in urban areas [29-31]. Moreover, living in low income area has positive association with diabetes (OR: 1.43) [28]. Economic status has negative association with diabetes, so low economic status and diabetes has OR: 1.52 [29]. According to mentioned studies, socioeconomic class was inversely associated by diabetes prevalence.

#### Diabetes control

There was a significant negative association between social capital score and diabetes control [33]. In this part, 4 articles included but the results were heterogeneous. In Isfahan, one study reported an association between gender and diabetes control (Female; OR: 1.56 and Male; 0.6) [33] . A national study showed this association; (Female; OR: 1.13 and Male; OR: 1) [34] and a study in Sanandaj showed male sex has OR: 2.46 related to HgA1c <6 in diabetes control [35].

A study considered age and marital status [36]. It revealed that age more than 55 years old have OR: 5.29 and single patients has OR: 0.94 with control of diabetes [34]. Another study showed that retired situation, unemployed, and housewife position accompanied with risk of uncontrolled diabetes (Their OR respectively were; 3.22, 2.59, 2.22) [33,35].

Regarding the educational level as a SEF, two study in Bandarabas and Sanandaj demonstrate illiteracy and diabetes control have OR: 1.24 and 3.42 [35,37] but in a national study, this association wasn’t seen (Illiterate; OR: 1 and literate; OR: 1.11) [34]. It is noticeable that, living in urban area and diabetes control had OR 1.39 [34].

#### Diabetes complications

Included studies showed significant relationship between diabetes complications frequency and age group [38,39]. A study demonstrated that, onset of diabetes under 45 years old is associated with cardiovascular disease (OR: 1.13) [40]. A study showed, complications’ frequency has significant relation with female sex and the other study revealed that male sex and cardiovascular disease probability in diabetes patients associated with OR: 1.79 [39,40]. Marital status as another SEF was associated with social functioning and general health domains but it is not associated with complications.

Educational level has significant association with number of complications so that more complications were more prevalent in higher education levels [38,39]. Also, occupation is related to diabetes complications. Housewives and retired people have the most number of complications [38]. Also, social class was effective factor and the most of patients belonged to low income group [38,39].

#### Other related subjects to diabetes

Among included studies, two cases were related to awareness of diabetes and food insecurity [41,42]. A study in Isfahan, Najafabad, and Arak revealed that, female sex, age of more than 30 years, educational levels under diploma, and retired situation have OR > 1 with awareness of diabetes [41].

Another study in Fars showed that, among diabetic patients, income and high economic status were protective factors of food insecurity but family size and number of children have OR more than three [42].

## Discussion

Our study has tried to cover all diabetes’ socioeconomic inequality studies in Iran in various domains; Inequality and diabetes, diabetes prevalence, diabetes control, diabetes complications, and other related subjects. Age, gender, educational level, occupation, income, and residence area were assessed in this regards [43].

We found an overall increase of diabetes prevalence among female sex, upper age groups, illiterate situation, retired and unemployed status, low economic condition and urban residency [28-31]. Similarly, several studies showed females and less educated persons are more exposed to diabetes [11,44-47]. Other studies indicate the most chronic disease is more prevalent in less wealthy people [48] and the diabetes prevalence is higher in low income people and retired person [46,49]. The mechanism of relation low socioeconomic position and diabetes are not clear. But, life style pattern may explain these differences [50].

There are controversial reports on association between some variables of SEFs such as educational level and diabetes control [33,35,36,51]. Some studies revealed positive association between educational level [35,37] and control of diabetes but the others have a reverse scenario [43]. Its inverse association also was seen in South Korea [43]. The reason may be that high level educated people are generally in young population and young people have lower treatment coverage [43]. Better control of diabetes in rural area might be due to successful primary health care (PHC) in Iran [43] and effective management of non-communicable disease by community health workers in rural area [52]. It is considerable that, retired position and unemployment situation have less diabetes control because their age and insurance condition exposed them to multi-morbidity and less treatment [33,35,44]. It is remarkable; most diabetic patients with complications were in the low income group [38,40]. Less diabetes control in this group could leads to more complications.

In present study we benefited from some power points; the comprehensive replicable study applied to international and national database with no limitation on time, age and language. Also, we considered restrictive method for quality assessment and data extraction. It should be noted that, this is the first systematic review about socioeconomic factors inequality and diabetes in Iran.

However, we faced to a few limitations. Among some included studies, required measures did not exist. The included studies were mostly heterogeneous. For that reason, we haven’t done meta-analysis and present results without statistical analysis.

Despite these limitations, we provided information could lead to the identification opportunities for health promotion in affected communities by inequitable conditions [53,54].

Evidences reveal that, evaluation of health inequalities, especially with focus on socioeconomic factors has been less intentioned in some developing countries [55]. According to Universal health coverage (UHC), strategic plan regarding health inequalities reduction is a duty of each country’s health system [56-58].

Considering above, the following suggestions proposed; Monitoring and evaluation of health care system regarding NCDs control, Promotion of Primary health care system in urban area, Primary prevention by lifestyle interventions especially in urban area, Applying Community based intervention programs [59,60]. Special attention to low socioeconomic class, Strategic planning to reduce disparity between provinces according to social, economic, and political differences [61,62], Health literacy promotion, improving general living conditions [63], and Providing the supportive resources and strategies.

## Conclusion

In conclusion, we found that diabetes prevalence is associated with socioeconomic factors and there is a need for appropriate policy making regarding social health determinants. Cost effective interventional programs would improve diabetes prevention, early diagnosis and appropriate treatment. Governments by financial support in poor areas and establish responsible insurance system could help to reduce the inequality. According to limited studies in our country, there is a strong need for further investigation regarding non-communicable diseases and social determinants of health at national and sub-national levels.

## References

[1] Global Burden of Disease. [http://vizhub.healthdata.org/gbd-compare/]

[2] The Global Burden of Disease, Guiding Policy Middle East and North Africa Regional Edition. [http://www.healthmetricsandevaluation.org/gbd/publications/policy-report/global-burden-disease-middle-east-north-africa]

[3] Lim SS, Vos T, Flaxman AD, Danaei G, Shibuya K, Adair-Rohani H, Amann M, Anderson HR, Andrews KG, Aryee M (2012). A comparative risk assessment of burden of disease and injury attributable to 67 risk factors and risk factor clusters in 21 regions, 1990-2010: a systematic analysis for the Global Burden of Disease Study 2010. Lancet.

[4] Danaei G, Finucane MM, Lu Y, Singh GM, Cowan MJ, Paciorek CJ, Lin JK, Farzadfar F, Khang Y-H, Stevens GA (2011). National, regional, and global trends in fasting plasma glucose and diabetes prevalence since 1980: systematic analysis of health examination surveys and epidemiological studies with 370 country-years and 2· 7 million participants. The Lancet.

[5] Li G, Zhang P, Wang J, An Y, Gong Q, Gregg E, Yang W, Zhang B, Shuai Y, Hong J (2014). Cardiovascular mortality, all-cause mortality, and diabetes incidence after lifestyle intervention for people with impaired glucose tolerance in the Da Qing Diabetes Prevention Study: a 23-year follow-up study. Lancet Diabetes Endocrinol.

[6] Hopper I, Billah B, Skiba M, Krum H (2011). Prevention of diabetes and reduction in major cardiovascular events in studies of subjects with prediabetes: meta-analysis of randomised controlled clinical trials. Eur J Cardiovasc Prev Rehabil.

[7] Collins, Anna L: Inequalities in global health: a world-system analysis, 1945-present. PhD thesis. Department of Sociology, Anthropology, and Social Work; 2013, Kansas State University.

[8] Joshi R, Jan S, Wu Y, MacMahon S (2008). Global inequalities in access to cardiovascular health care: our greatest challenge. J Am Coll Cardiol.

[9] Nandi A, Glymour MM, Subramanian SV (2014). Association among socioeconomic status, health behaviors, and all-cause mortality in the United States. Epidemiology (Cambridge, Mass).

[10] Bachmann M, Eachus J, Hopper C, Davey SG, Propper C, Pearson N, Williams S, Tallon D, Frankel S (2003). Socio-economic inequalities in diabetes complications, control, attitudes and health service use: a cross-sectional study. Diabet Med.

[11] Espelt A, Kunst A, Palència L, Gnavi R, Borrell C (2012). Twenty years of socio-economic inequalities in type 2 diabetes mellitus prevalence in Spain, 1987-2006. Eur J Public Health.

[12] Socio-economic-determinants-of-health [http://www.idf.org/socio-economic-determinants-of-health].

[13] Agardh E, Allebeck P, Hallqvist J, Moradi T, Sidorchuk A (2011). Type 2 diabetes incidence and socio-economic position: a systematic review and meta-analysis. Int J Epidemiol.

[14] Saydah SH, Imperatore G, Beckles GL (2013). Socioeconomic Status and Mortality: Contribution of health care access and psychological distress among US adults with diagnosed diabetes. Diabetes Care.

[15] Mackenbach, J. P., Meerding, W. J., & Kunst, A. Economic implications of socio-economic inequalities in health in the European Union. European Commission .2007. http://ec.europa.eu/health/ph_determinants/socio_economics/documents/socioeco_inequalities_en.pdf. Accessed 15 Dec 2014.

[16] Jepson RG, Harris FM, Platt S, Tannahill C (2010). The effectiveness of interventions to change six health behaviours: a review of reviews. BMC Public Health.

[17] Kotwani N, Danis M (2009). Expanding the Current Health Care Reform Debate: Making the Case for Socio-Economic Interventions for Low Income Young Adults. Health Care L & Policy.

[18] Kontis V, Mathers CD, Rehm J, Stevens GA, Shield KD, Bonita R, Riley LM, Poznyak V, Beaglehole R, Ezzati M (2014). Contribution of six risk factors to achieving the 25x25 non-communicable disease mortality reduction target: a modelling study. Lancet.

[19] Global monitoring framework. [http://www.who.int/nmh/global_monitoring_framework/en/]

[20] Hsu C-C, Lee C-H, Wahlqvist ML, Huang H-L, Chang H-Y, Chen L, Shih S-F, Shin S-J, Tsai W-C, Chen T (2012). Poverty Increases Type 2 Diabetes Incidence and Inequality of Care Despite Universal Health Coverage. Diabetes Care.

[21] Connolly V, Unwin N, Sherriff P, Bilous R, Kelly W (2000). Diabetes prevalence and socioeconomic status: a population based study showing increased prevalence of type 2 diabetes mellitus in deprived areas. J Epidemiol Community Health.

[22] Brown AF, Ettner SL, Piette J, Weinberger M, Gregg E, Shapiro MF, Karter AJ, Safford M, Waitzfelder B, Prata PA (2004). Socioeconomic position and health among persons with diabetes mellitus: a conceptual framework and review of the literature. Epidemiol Rev.

[23] STEPs reports. [http://www.ncdinfobase.ir/]

[24] Diabetes melitus [http://www.ncbi.nlm.nih.gov/mesh/68003920].

[25] Socioeconomic factors. [http://www.ncbi.nlm.nih.gov/mesh/68012959]

[26] CASP checklists. [http://www.casp-uk.net/#!casp-tools-checklists/c18f8]

[27] Emamian MH, Alami A, Fateh M (2011). Socioeconomic inequality in non-communicable disease risk factors in Shahroud, Iran. Iranian J Epidemiology.

[28] Maddah M (2010). Association of diabetes with living area in Iranian women. Int J Cardiol.

[29] Golozar A, Khademi H, Kamangar F, Poutschi H, Islami F, Abnet CC, Freedman ND, Taylor PR, Pharoah P, Boffetta P (2011). Diabetes Mellitus and Its Correlates in an Iranian Adult Population. PLoS One.

[30] Gholamreza V, Hamidreza J, Ahmad H, Abdolhamid Angizeh E, Tazik PMM (2010). Assessment of Diabetes Mellitus type II and some Related Factors among Adult People aged 25-65 Years old in Golestan Province, Iran. Journal of Gorgan Bouyeh Faculty of Nursing & Midvifery.

[31] Azimi-Nezhad M, Ghayour-Mobarhan M, Parizadeh MR, Safarian M, Esmaeili H, Parizadeh SM, Khodaee G, Hosseini J, Abasalti Z, Hassankhani B, Ferns G (2008). Prevalence of type 2 diabetes mellitus in Iran and its relationship with gender, urbanisation, education, marital status and occupation. Singapore Med J.

[32] Shahraki M, Shahraki T, Shidfar F, Ansari H (2012). Which modifiable, non-modifiable, and socioeconomic factors have more effect on cardiovascular risk factors in overweight and obese women?. J Res Med Sci.

[33] Farajzadegan Z, Jafari N, Nazer S, Keyvanara M, Zamani A (2013). Social capital–a neglected issue in diabetes control: a cross‐sectional survey in Iran. Health Soc Care Community.

[34] Mirzazadeh A, Baradaran HR, Haghdoost AA, Salari P (2009). Related factors to disparity of diabetes care in Iran. Med Sci Monit.

[35] Esmaeil-Nasab N, Abdolrahimzadeh A, Ebrahimi A (2010). The cross-sectional study of effective factors on type 2 diabetes control in a diabetes care center in Sanandaj. Iranian Epidemiology Journal.

[36] Mirzazadeh A, Baradaran HR, Haghdoost AA, Salari P (2009). Related factors to disparity of diabetes care in Iran. Med Sci Monit.

[37] Jahanlou AS, Alishan Karami N (2011). The effect of literacy level on health related-quality of life, self-efficacy and self-management behaviors in diabetic patients. Acta Med Iran.

[38] Tol A, Sharifirad G, Shojaezadeh D, Tavasoli E, Azadbakht L (2013). Socio-economic factors and diabetes consequences among patients with type 2 diabetes. J Educ Health Promot.

[39] Tol A, Pourreza A, Shojaeezadeh D, Mahmoodi M, Mohebbi B (2012). The assessment of relations between socioeconomic status and number of complications among type 2 diabetic patients. Iran J Public Health.

[40] Rahimian BE (2011). Risk factors for cardiovascular complications in patients with type II diabetes; predictive role of psychological factors, social factors and disease charactristics. Quar j Fund Mental Health.

[41] Shirani S, Kelishadi R, Sarrafzadegan N, Khosravi A, Sadri G, Amani A, Heidari S, Ramezani MA (2009). Awareness, treatment and control of hypertension, dyslipidaemia and diabetes mellitus in an Iranian population: the IHHP study. East Mediterr Health J.

[42] Najibi N, Dorosty Motlagh A, Sadrzadeh Yeganeh H, Eshraghian M, Daneshi M, Azizi S. Food insecurity status and some associated socioeconomic factors among newly diagnosed patients with type 2 diabetes in Shiraz, 2012**.** AMUJ 1392, 16**:**98**–**106.

[43] Di Cesare M, Khang YH, Asaria P, Blakely T, Cowan MJ, Farzadfar F, Guerrero R, Ikeda N, Kyobutungi C, Msyamboza KP (2013). Inequalities in non-communicable diseases and effective responses. The Lancet.

[44] Boutayeb A, Boutayeb S, Boutayeb W (2013). Multi-morbidity of non communicable diseases and equity in WHO Eastern Mediterranean countries. Int J Equity Health.

[45] Imkampe AK, Gulliford MC (2011). Increasing socio-economic inequality in type 2 diabetes prevalence—Repeated cross-sectional surveys in England 1994–2006. Eur J Public Health.

[46] Shamshirgaran SM, Jorm L, Bambrick H, Hennessy A. Independent roles of country of birth and socioeconomic status in the occurrence of type 2 diabetes. BMC public health 2013;13. 10.1186/1471-2458-13-1223 PMC3883476 24359144

[47] Babaie MH: Inequities in health and health care between provinces of Iran: promoting equitable health care resource allocation .2012.http://usir.salford.ac.uk/30807/. Accessed 15 Dec 2014

[48] Sozmen K, Belgin U (2014). Socioeconomic Inequalities in Non-Communicable Diseases and Self Assessed Health in Turkey. Iran J Public Health.

[49] Hosseinpoor AR, Bergen N, Mendis S, Harper S, Verdes E, Kunst A, Chatterji S (2012). Socioeconomic inequality in the prevalence of noncommunicable diseases in low-and middle-income countries: Results from the World Health Survey. BMC Public Health.

[50] Ng M, Fleming T, Robinson M, Thomson B, Graetz N, Margono C, Mullany EC, Biryukov S, Abbafati C, Abera SF (2014). Global, regional, and national prevalence of overweight and obesity in children and adults during 1980–2013: a systematic analysis for the Global Burden of Disease Study 2013. The Lancet.

[51] Gakidou E, Mallinger L, Abbott-Klafter J, Guerrero R, Villalpando S, Ridaura RL, Aekplakorn W, Naghavi M, Lim S, Lozano R, Murray CJ (2011). Management of diabetes and associated cardiovascular risk factors in seven countries: a comparison of data from national health examination surveys. Bull World Health Organ.

[52] Farzadfar F, Murray CJL, Gakidou E, Bossert T, Namdaritabar H, Alikhani S, Moradi G, Delavari A, Jamshidi H, Ezzati M (2012). Effectiveness of diabetes and hypertension management by rural primary health-care workers (Behvarz workers) in Iran: A nationally representative observational study. The Lancet.

[53] M B (2011). Relationship between Economic Growth, Income Inequality and Health in Iran: 1978–2006. Iranian J Epidemiology.

[54] Emadzadeh M, Samadi S, Paknezhad S. The Impact of Inequal Distribution of Income on the Health Status in Selected Organization of Islamic Countries (OIC). Journal of Health Information Management, 8:306–314.

[55] M V, A M (2006). Socio-Economic Health Inequalities and their Indices in Epidemiologic Studies. Iranian Journal of Epidemiology.

[56] McCarthy M (2013). Reducing inequality is crucial to implementing universal health coverage, says WHO report. BMJ.

[57] Universal Health Coverage. [http://www.who.int/health_financing/universal_coverage_definition/en/]

[58] Albert MA, Ayanian JZ, Silbaugh TS, Lovett A, Resnic F, Jacobs A, Normand SL (2014). Early results of Massachusetts healthcare reform on racial, ethnic, and socioeconomic disparities in cardiovascular care. Circulation.

[59] Kelishadi R, Poursafa P (2014). A review on the genetic, environmental, and lifestyle aspects of the early-life origins of cardiovascular disease. Curr Probl Pediatr Adolesc Health Care.

[60] Majeed A, El-Sayed AA, Khoja T, Alshamsan R, Millett C, Rawaf S (2014). Diabetes in the Middle-East and North Africa: An update. Diabetes Res Clin Pract.

[61] Astell-Burt T, Feng X, Kolt GS, McLean M, Maberly G (2014). Understanding geographical inequities in diabetes: Multilevel evidence from 114,755 adults in Sydney, Australia. Diabetes Res Clin Pract.

[62] Akhgary M, Tabatabaee SM, Bromand MG, Amiri MA (2010). Trends of alterations in disparities of mortality in rural areas of different provinces (Iran, 1993–2008). Scientific Journal of Kurdistan University of Medical Sciences.

[63] Klijs B, Nusselder WJ, Looman CW, Mackenbach JP (2014). Educational Disparities in the Burden of Disability: Contributions of Disease Prevalence and Disabling Impact. Am J Public Health.

